# Spontaneous coronary artery dissection ‘storm’ in post-partum: a case report

**DOI:** 10.1093/ehjcr/ytae567

**Published:** 2024-10-26

**Authors:** Nicolas Combaret, Géraud Souteyrand, Pascal Motreff

**Affiliations:** Department of Cardiology, CHU Clermont-Ferrand, CNRS, Université Clermont Auvergne, 58 rue Montalembert, 63000 Clermont-Ferrand, France; Department of Cardiology, CHU Clermont-Ferrand, CNRS, Université Clermont Auvergne, 58 rue Montalembert, 63000 Clermont-Ferrand, France; Department of Cardiology, CHU Clermont-Ferrand, CNRS, Université Clermont Auvergne, 58 rue Montalembert, 63000 Clermont-Ferrand, France

**Keywords:** Acute coronary syndrome, Spontaneous coronary artery dissection (SCAD), Optical coherence tomography (OCT), Case report

## Abstract

**Background:**

Spontaneous coronary artery dissection (SCAD) remains a rare form of acute coronary syndrome (ACS) in young women. It is not always easy to diagnose and its management can be complex, particularly in the case of severe forms.

**Case summary:**

A 29-year-old Mahorese woman presented with non-ST-elevation acute coronary syndrome 72 h after vaginal delivery of an uncomplicated twin pregnancy. Coronary angiography revealed a SCAD of the left anterior descending artery (LAD) treated medically. At Day 4, urgent coronary angiography was performed due to recurrent ACS with unfavourable evolution on LAD SCAD with worsening flow and appearance of right coronary artery (RCA) dissection. Percutaneous coronary intervention (PCI) was performed with drug-eluting stent (DES) implantation in LAD under optical coherence tomography (OCT) guidance. At Day 6, the patient suffered from inferior ST segment elevation myocardial infarction due to extension of the RCA SCAD with occlusion of the posterior descending artery. Once again, under OCT guidance, PCI was performed in RCA with implantation of four DES. At Day 8, the patient presented with an intra-hospital cardiac arrest by electromechanical dissociation. Urgent coronary angiography revealed a diffuse extension of the SCAD to the circumflex artery and the left main trunk (LM). Rescue PCI of the LM was performed under OCT guidance, and a femoro-femoral extracorporeal life support was implanted because of haemodynamic instability. With no hope of left ventricular ejection fraction recovery, the patient eventually benefited from a heart transplant 12 days after the first ACS with excellent evolution.

**Discussion:**

Although the majority of SCAD have a favourable outcome with spontaneous healing, it is important to keep in mind that certain severe forms exist and could require specific management as PCI under OCT guidance and potential transfer to surgical centres where heart transplants and mechanical circulatory support are available.

**Learning points**

- Spontaneous coronary artery dissection (SCAD) can have a catastrophic outcome in the peripartum setting. Even if conservative treatment is preferred and percutaneous coronary intervention is performed under intra-coronary imaging guidance, some of these cases have an unfavourable outcome.
- In complex cases of SCAD, transfer to a cardiac surgery centre for mechanical circulatory support or heart transplantation may be proposed.

## Introduction

Spontaneous coronary artery dissection (SCAD) remains a rare form of acute coronary syndrome. It affects young women with an average age of 50 and few cardiovascular risk factors.^1^ Diagnosis is not always evident, as some angiographic forms mimic atherosclerotic lesions. Certain angiographic signs are known,^2^ and SCAD lesions can therefore be classified.^3^ Medical treatment should be favoured and can be carried out in the majority of cases. However, percutaneous coronary intervention (PCI) is sometimes necessary [poor thrombolysis in myocardial infarction (TIMI) flow, haemodynamic instability, persistent chest pain] with a high complication rate.^4,5^ Intra-coronary imaging [intravascular ultrasound or optical coherence tomography (OCT)], which can help with diagnosis, can also be a valuable tool for ensuring the safety of the PCI procedure (for example, ensuring that the guide wire is in the true lumen).

## Summary figure

**Figure:**
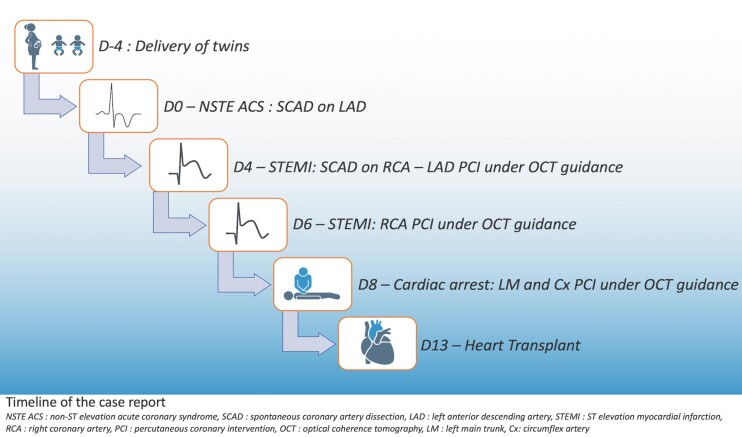


## Case presentation

We report the case of a 29-year-old woman admitted to our unit for non-ST-elevation acute coronary syndrome 72 h after vaginal delivery of an uncomplicated twin pregnancy. She had obesity as a cardiovascular risk factor. She had no medical history and this was her fourth pregnancy (including one miscarriage). She developed gestational diabetes during this pregnancy, and the delivery was marked by pre-eclampsia syndrome treated with labetalol.

**Figure 1:**
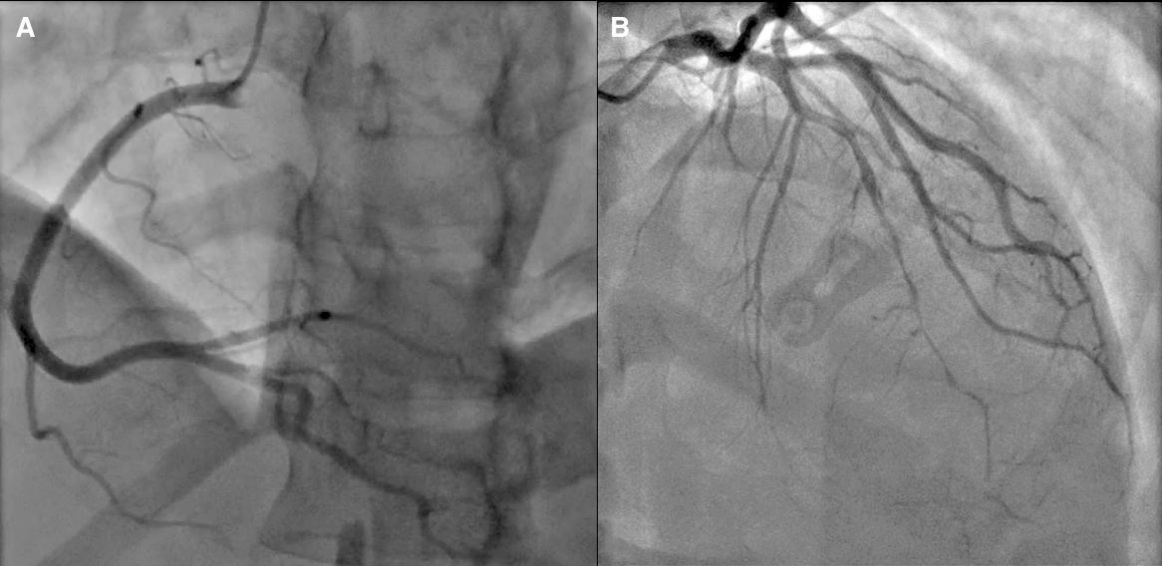
Initial angiogram at D0 revealing a normal right coronary artery (RCA) (*A*) and an extensive spontaneous coronary artery dissection (SCAD) affecting the left anterior descending artery (LAD) with true dissection in the mid part and compressive haematoma in the distal part (*B*).

On admission, the clinical examination was normal and there was no chest pain. The electrocardiogram and transthoracic echocardiography (TTE) were unremarkable, but the troponin level was positive. In this setting, the patient underwent coronary angiography which revealed a spontaneous left anterior descending coronary artery (LAD) dissection with an intimal flap in the mid part and a long distal haematoma (*Figure 1*).

Due to the absence of chest pain and ECG changes with TIMI 3 flow, it was decided to treat this SCAD medically with single antiplatelet therapy and betablockers.

**Figure 2:**
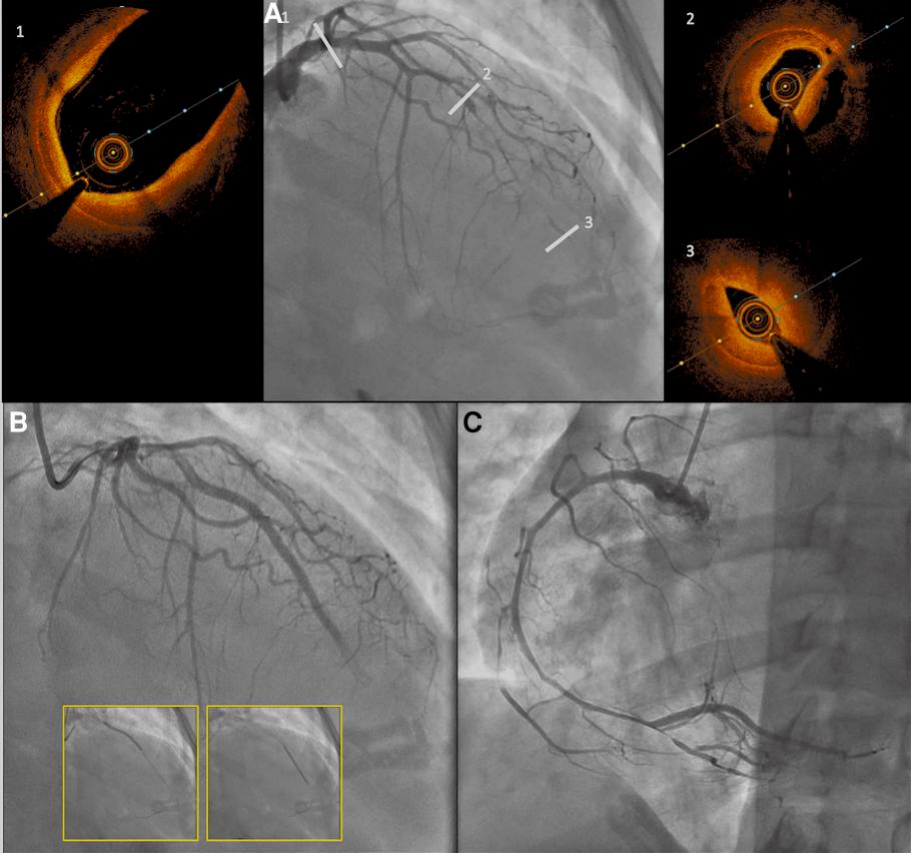
Control angiography at D4 revealing a worsening of the spontaneous coronary artery dissection (SCAD) on left anterior descending artery (LAD) with TIMI1 flow (*A*). Optical coherence tomography (OCT) revealed an extension of the haematoma on the left main coronary artery (1), a circulating haematoma in the mid part (2), and a voluminous compressive haematoma on the distal part of the LAD (3). PCI was performed under optical coherence tomography (OCT) guidance by implanting two drug-eluting stents (*B*). The angiographic control of the right coronary artery (RCA) showed an extensive SCAD with TIMI 3 flow (*C*).

While the patient was still in the intensive care unit, she presented with new chest pain on the fourth day with negative T-wave changes on the ECG. An angiographic control was performed in emergency: the evolution was unfavourable on the LAD with worsening TIMI flow (*Figure 2*). The right coronary artery (RCA), which was normal 4 days previously, revealed a long dissection with a short intimal dissection on its proximal part and a long haematoma extending to the heart crux with a TIMI 3 flow (*Figure 2*). Regarding the TIMI 1 flow on LAD, it was decided to perform PCI guided by OCT imaging (*Figure 2*) and to treat the RCA dissection medically. Optical coherence tomography confirms an extensive haematoma from the proximal to the distal part of the LAD without affecting the left main trunk. After confirmation of the position of the guide wire in the true lumen, scoring balloon (NSE Alpha 2.0 × 13 mm) was inflated and two drug-eluting stents (DESs) were implanted (SYNERGY 2.5 × 38 mm in distal and 2.75 × 38 mm in proximal part). The immediate angiographic result was not satisfactory due to the persistence of a slowed TIMI flow in the distal part of the LAD but the patient was relieved and the ECG improved. No additional OCT was performed after PCI. After procedure, TTE revealed apical akinesia with left ventricular ejection fraction (LVEF) at 55%.

**Figure 3:**
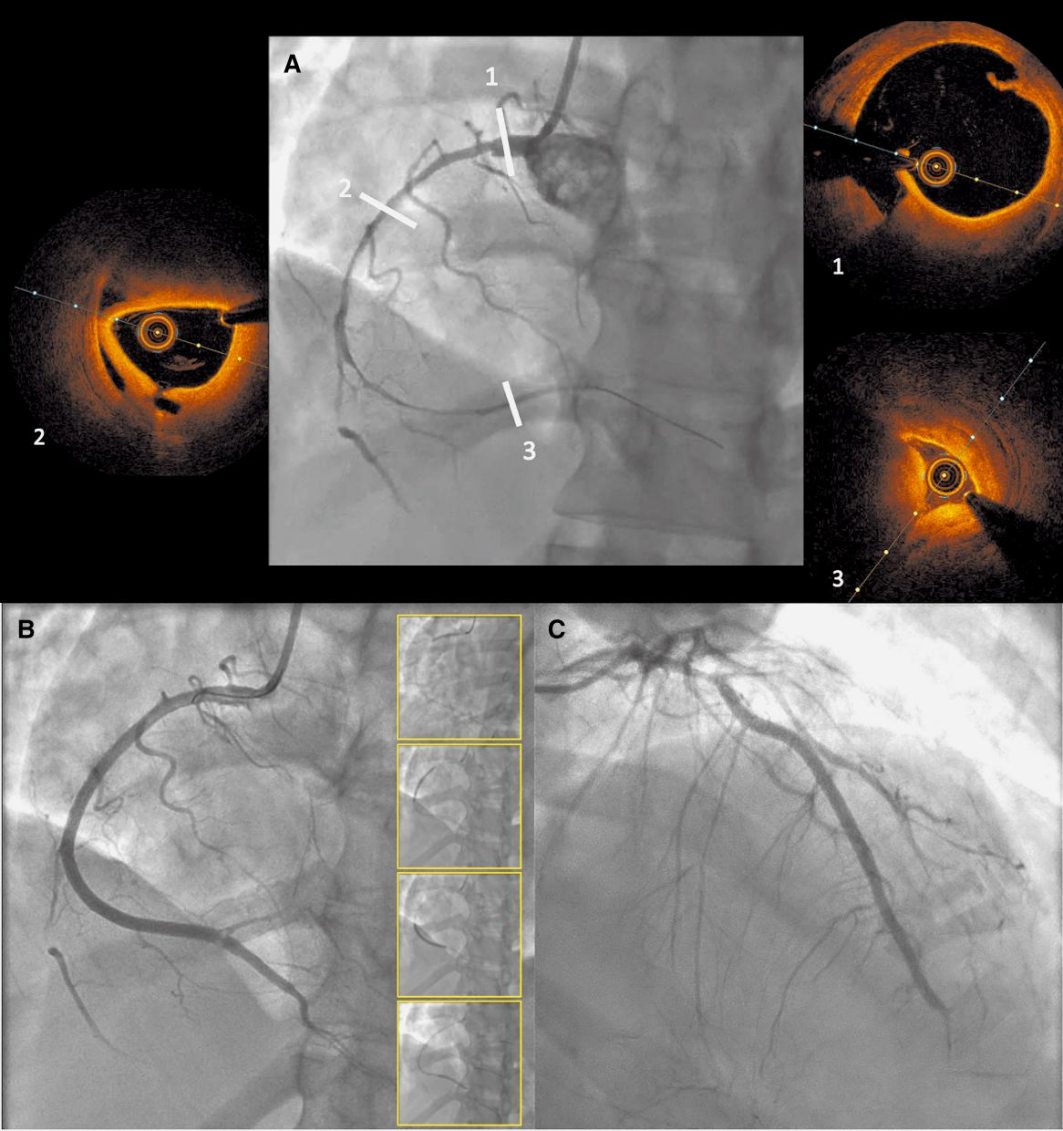
Control angiography at D6 in context of inferior ST-elevation myocardial infarction STEMI. The evolution on the right coronary artery (RCA) was not favourable with worsening of the haematoma and occlusion of the posterior descending artery (PDA) (*A*). Optical coherence tomography (OCT) revealed the initial intimal rupture (1), two lumens in the mid part and compressive haematoma in the distal part of the RCA. Percutaneous coronary intervention (PCI) was performed in emergency with implantation of four drug-eluting stents (*B*). Control angiogram of the left anterior descending artery (LAD) revealed an extension of the haematoma in the proximal part (*C*).

To avoid new angiographic control with high risk of iatrogenic dissection, CT scan was programmed to assess the RCA SCAD but 4 days after the LAD PCI, the patient presented with an inferior ST segment elevation myocardial infarction (STEMI). Emergency angiography revealed a non-compressive haematoma of the left main artery, the proximal LAD artery, and the entire circumflex coronary artery. The PCI result was acceptable with TIMI 3 flow but persistent occlusion of the distal LAD. The posterior descending artery (PDA) was now occluded with TIMI 2 flow in the posterolateral artery (*Figure 3*). Once again, PCI was performed under OCT guidance. Optical coherence tomography imaging confirmed an intimal tear in the proximal part and the correct position of the guidewire to distality (*Figure 3*). After scoring balloon dilatation, four DES from proximal RCA to PDA were implanted with RePOT (Proximal Optimization Technique) bifurcation technique at the heart crux. Immediate angiographic result was satisfactory but the posterolateral remained dissected. The electrocardiogram improved and the chest pain disappeared. On post-PCI TTE, LVEF fell to 45% and an apical thrombus developed, requiring anticoagulant therapy in addition to dual antiplatelet therapy.

**Figure 4:**
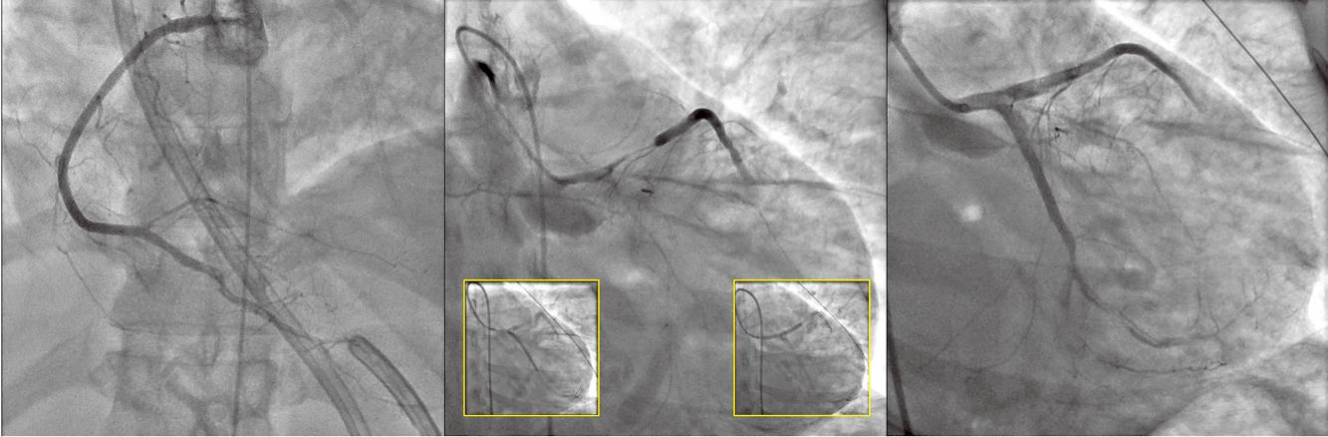
Control angiogram at D8 after cardiac arrest. The result of the right coronary artery (RCA) percutaneous coronary intervention (PCI) was correct (*A*) but the haematoma was now compressive in the left main trunk, the proximal left anterior descending artery (LAD), and the proximal circumflex artery (*B*). Angiogram after rescue PCI is shown in *C*.

Unfortunately, 2 days after the second PCI, the patient presented an intra-hospital cardiac arrest (electromechanical dissociation). Cardiopulmonary resuscitation was initiated immediately, with a no-flow period of a few seconds. After administration of 1 mg of adrenaline, spontaneous circulation was restored. The low-flow period lasted 7 min. A femoro-femoral extracorporeal life support was implanted because of haemodynamic instability. Emergency coronary angiography was performed and revealed a catastrophic evolution with an extension of the haematoma to all the left coronary artery (*Figure 4*). Percutaneous coronary intervention under OCT guidance was attempted with left main PCI: scoring balloons were inflated in the left main and in the proximal LAD and circumflex to restore a flow. A DES was finally implanted in the left main to the circumflex with RePOT bifurcation technique (*Figure 4*). Transthoracic echocardiography post-PCI showed a LVEF < 10% with no aortic valve opening under extra corporeal life support. With no hope of LVEF recovery and after clinical stabilization, the patient underwent heart transplant 5 days after her cardiac arrest with excellent evolution.

At 2 years, the patient remains asymptomatic. Left ventricular ejection fraction is normal with no signs of rejection.

## Discussion

Spontaneous coronary artery dissection has long been considered a peripartum disease. Recent data suggest that this condition affects young women with few or no cardiovascular risk factors,^1,6^ but hormonal status does not appear to be a favourable factor. According to recent data, the prevalence of SCAD in the peripartum period is estimated between 4.4% and 17% of cases^5,7^. It is possible, however, that peripartum forms are more severe (poor left ventricle ejection fraction, left main or multi vessel SCAD).^8^

Conservative management is recommended in cases of clinical stability (haemodynamics and no residual chest pain) and TIMI 3 flow, with a good outcome in the majority of cases.^9^ We present here the case of a post-partum SCAD with an unfavourable short-term outcome despite an initial conservative strategy. We also recall the difficulty of coronary angioplasties in the context of SCAD and the need to perform them under OCT guidance to confirm the presence of the guide wire in the true lumen.^4,5^ Our strategy of guided OCT PCI did not lead to a favourable outcome, but it did enable the patient to be stabilized and, as a last resort, to consider a heart transplant under better conditions. What could have been done differently to hope for a favourable outcome? During the urgent angiographic control at D4, we could have treated the RCA dissection with PCI under OCT guidance to avoid the pejorative evolution we observed. However, this would probably not have prevented the poor evolution of the LAD and Cx favoured by the anticoagulant treatment introduced for the apical thrombus.

In the light of this case, we can make the following recommendations: (i) always carry out PCI in the context of SCAD under OCT; (ii) in the event of an unfavourable initial course, it would seem appropriate on the one hand to consider close monitoring in the ICU and possibly to propose monitoring of the SCAD by non-invasive imaging (CT scan) and on the other hand to consider a transfer to a centre with cardiac surgery in the event of the need for mechanical circulatory support; and (iii) in the case of a cataclysmic evolution such as ours, mechanical circulatory support sometimes in bridge to a heart transplant is a viable option.

## Lead author biography


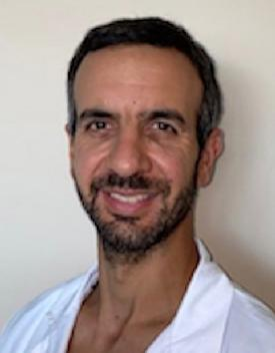
Dr Nicolas Combaret is a French interventional cardiologist. His areas of research include acute coronary syndrome, in particular spontaneous coronary artery dissection (SCAD), and intra-coronary imaging.

##  

**Consent:** The authors confirm that written consent for submission and publication of this case report has been obtained from the patient in line with COPE guidelines.

**Funding:** None.

## Data Availability

The data underlying this article will be shared on reasonable request to the corresponding author.
